# Enhancing Chronic Disease Management: Personalized Medicine Insights from Rural and Urban General Practitioner Practices

**DOI:** 10.3390/jpm14070706

**Published:** 2024-07-01

**Authors:** Marta Duda-Sikuła, Donata Kurpas

**Affiliations:** 1 Clinical Trial Support Centre, Wroclaw Medical University, 50-367 Wrocław, Poland; 2 Department of Family and Pediatric Nursing, Faculty of Health Sciences, Wroclaw Medical University, 50-996 Wrocław, Poland; donata.kurpas@umw.edu.pl

**Keywords:** chronicdisease, prevention and control, diabetes, hypertension, personalized medicine

## Abstract

Health policies worldwide emphasize managing chronic conditions like diabetes and hypertension through medication and lifestyle modifications. However, translating guidelines into practical application remains challenging, leading to suboptimal care and poor health outcomes, particularly in low-resource settings. This study aims to reveal significant differences between rural and urban patients requiring personalized approaches to chronic disease management based on geographical location and demographic data, considering the impact of emergencies such as the COVID-19 pandemic. Data were collected from rural and urban general practitioner (GP) practices in Poland, covering four years from 2018 to the first quarter of 2021, focusing on diabetes and hypertension epidemiology, risk factors, comorbidities, resource consumption, and disease burden. The findings revealed significant differences between rural and urban patients regarding age, number of patient visits, gender distribution, and types of diagnoses and visit modalities. Rural patients tended to be older, had a higher median number of visits, and exhibited different patterns of diagnoses and visit types compared to urban patients. The study also investigated the impact of the COVID-19 pandemic on chronic disease treatment, finding that while age at visits increased during the pandemic, there were no significant changes in gender distribution, but a noticeable shift in diagnoses and visit modalities with an increase in remote visits and changes in the prevalence of specific diagnoses. These disparities highlight the need for tailored approaches to chronic disease management based on geographic location and patient demographics. The study underscores the importance of understanding the unique challenges and opportunities in managing chronic diseases across different settings and during public health crises like the COVID-19 pandemic, aiding healthcare providers and policymakers in developing targeted interventions to improve chronic disease prevention and management, ultimately leading to better health outcomes for individuals and communities. Further research is needed to explore the long-term effects of the pandemic on chronic disease treatment and assess the effectiveness of interventions to mitigate its impact.

## 1. Introduction

Health policies today prioritize managing chronic conditions through medication and lifestyle modifications [1]. Primary healthcare (PHC) focuses on preventing chronic conditions by engaging patients and ensuring equitable access to care [2]. More than merely providing advice is required; patients require well-implemented guidelines for effective self-management [3]. However, existing guidelines often need to translate into practical application, hindering optimal care and contributing to poor health outcomes [4]. Thus, there isa need for evidence-based, practical guidelines tailored to diverse patient needs [5]. Key challenges include integrating self-management into clinical practice and addressing health disparities, particularly in rural areas [6,7]. Personalized interventions within guidelines and supporting lifestyle changes remain significant challenges [8,9].

Non-communicable diseases (NCDs), including cardiovascular diseases, cancers, chronic respiratory diseases, and diabetes, affect individuals worldwide and are attributed to various risk factors such as unhealthy diets, physical inactivity, tobacco use, and excessive alcohol consumption [1]. These diseases contribute to a significant portion of global mortality, with NCD-related deaths occurring across all age groups and regions, particularly impacting low- and middle-income countries [1]. The World Health Organization (WHO) has outlined a Global Action Plan for the Prevention and Control of NCDs, emphasizing population-level interventions like promoting healthy consumption and individual-level interventions such as cardio-metabolic risk assessment and management [1,10]. However, countries must periodically evaluate their progress in implementing evidence-based guidelines to effectively address NCDs and prioritize interventions tailored to their contexts [1,10]. Efforts should focus on understanding the evolving factors contributing to the NCD burden across different regions [1,10].

Hypertension and diabetes represent significant global public health challenges, with millions affected worldwide, particularly in low- and middle-income countries [11,12]. Hypertension prevalence varies regionally, with Africa experiencing the highest rates, contributing significantly to cardiovascular disease and premature mortality [12]. Efforts to address hypertension and diabetes include promoting healthy lifestyles, enhancing treatment access, and implementing effective management strategies [13,14,15]. Effective chronic disease prevention and control strategies involve evidence-based practices, patient-centered approaches, self-management support, interdisciplinary teamwork, and capacity-building initiatives [16,17,18,19,20]. However, challenges remain in developing comprehensive, culturally sensitive guidelines to improve outcomes in low-resource settings [21,22,23].

Poland’s preventable mortality rates remain high despite improvements in cardiac care and cancer screening, with healthcare quality and patient safety still needing enhancement [24,25]. Preventive care spending is low, and risk factors like tobacco, alcohol, and air pollution significantly impact mortality. The 2015 Act on Public Health and the 2021–2025 National Health Programme aim to address issues like obesity, mental health, and environmental risks [26]. Life expectancy is below the EU average, with gender disparities and healthcare worker shortages, especially in rural areas, contributing to inequalities in health outcomes by income, age, and location [26].

In Poland, significant inequalities persist across various domains of health. Educational disparities are evident, with men with the lowest levels of education living on average 12 years less than those with tertiary education, while for women, the gap is 5.1 years [26]. Geographical disparities in life expectancy and mortality rates are also notable, particularly in the Łódzkie voivodeship, where the worst results are observed [26]. Efforts to reduce these inequalities include infrastructure investments, particularly in eastern regions [26]. Chronic diseases contribute substantially to the burden of illness, with nearly two in five adults in Poland reporting at least one chronic condition [26] and hypertension and diabetes representing significant health risks [27,28]. Hypertension control remains low compared to high-income countries [20,29], while diabetes prevalence has increased globally [30]. Cardiovascular diseases, particularly ischemic heart disease, are the leading causes of mortality, resulting in Poland having shorter life expectancies compared to most EU countries [26]. Inequalities in access to health policy programs and risk factors such as smoking, obesity, and physical inactivity contribute to disparities in health outcomes, with education and income playing significant roles [26]. Targeted interventions are necessary to address these disparities and improve public health outcomes in Poland.

Non-pharmacological approaches are crucial in managing hypertension, encompassing lifestyle modifications like weight management, dietary changes, physical activity, smoking cessation, alcohol moderation, and stress management [31]. These interventions effectively reduce blood pressure levels and mitigate cardiovascular risks, making them essential components alongside pharmacotherapy, especially in high-risk patients [32,33]. Lifestyle modifications involve achieving and maintaining a healthy body weight, adopting a balanced diet rich in fruits and vegetables while reducing salt and alcohol intake, engaging in regular physical activity, quitting smoking, and implementing stress-reduction techniques like meditation and deep breathing exercises [34]. These strategies collectively contribute to better blood pressure control and cardiovascular health, complementing pharmacological treatments [31].

Primary healthcare systems prevent and manageNCDs such as hypertension and diabetes [35]. Primary healthcare providers identify at-risk individuals through early detection, regular screenings, and diagnosis and initiate interventions to prevent or delay NCD onset [36]. Once diagnosed, primary healthcare facilitates effective management through medical treatment, lifestyle counseling, patient education, and support [37]. Health promotion and disease prevention initiatives further empower communities to adopt healthy behaviors, ultimately reducing the burden of NCDs and improving population health outcomes [38,39]. By integrating these comprehensive approaches, primary healthcare systems can significantly mitigate the impact of NCDs and enhance overall well-being at individual and community levels.

The COVID-19 pandemic has profoundly affected post-diagnostic care for individuals with chronic diseases, leading to decreased access to essential health services and disruptions in care delivery [40]. Studies published in *The Lancet Digital Health* and the *Journal of Medical Internet Research* underscore the decline in care utilization among patients with chronic conditions during the pandemic, highlighting the urgent need for healthcare systems to mitigate these impacts and ensure uninterrupted care provision [41,42]. Reports from the World Health Organization emphasize the necessity of adapting and strengthening healthcare systems to address these challenges and maintain quality care for individuals with chronic diseases despite the pandemic [8]. This disruption in care delivery underscores the importance of implementing strategies like telemedicine to facilitate remote care delivery. However, challenges such as limited technology access and quality concerns still need to be addressed [42]. Efforts are required to address these challenges and safeguard the health and well-being of individuals with chronic diseases during the pandemic and post-pandemic era [40].

### Objectives

This study aims to reveal significant differences between rural and urban patients requiring personalized approaches to chronic disease management based on geographical location and demographic data. This analysis also considers the impact of emergencies such as the COVID-19 pandemic on these differences. We conducted a retrospective case study of rural and urban general practitioner (GP) practices in Poland, focusing on diabetes and hypertension epidemiology, risk factors, comorbidities, resource consumption, and disease burden.

## 2. Materials and Methods

Study Design: The study design involves a retrospective analysis of data collected before and during the COVID-19 pandemic to assess its potential impact on the variables studied. Data were collected from rural and urban GP practices’ databases covering four years spanning 2018 to the first quarter of 2021. The pre-COVID period included data collected until 29 February 2020, while the COVID period comprised data collected from 1 March 2020 onward. The analysis was conducted separately for rural and urban subgroups to discern unique challenges and opportunities in managing diabetes and hypertension across different settings.

Setting: The urban primary healthcare center analyzed is located in the fourth-largest city in Poland, catering to approximately 4000 patients with 21,700 visits in 2022. In contrast, the rural primary healthcare center selected serves a small village in southern Poland with around 1200 residents, receiving approximately 11,000 patient visits in 2022. Both centers utilized the same software for clinic management, facilitating data unification and analysis.

Participants: The study included 13,833 patient visits for individuals diagnosed with diabetes (ICD-10 codes: E10 for Type 1 diabetes mellitus, E11 for Type 2 diabetes mellitus) and hypertension (ICD-10 codes: I10 for Essential Hypertension, I11 for Hypertensive heart disease with heart failure). Patient visits were recorded over the specified four-year period, allowing for comprehensive analysis before, during, and after the COVID-19 pandemic.

Data Sources/Measurement: Data were obtained from anonymized patient records, including demographic information, diagnoses, examination findings, and recommendations. The analysis utilized the same software for clinics in rural and urban centers, ensuring data collection and management consistency.

Bias: Efforts were made to address potential sources of bias by anonymizing patient data and ensuring data consistency across centers. The study received ethical approval from the Bioethics Committee at the Medical University of Wroclaw.

Study Size: The study size was determined based on the available data from rural and urban GP practices over the specified four-year period, aiming to provide a comprehensive sample for analysis.

Statistical Methods: Statistical analyses included the Wilcoxon test to assess quantitative variables across qualitative categories and Fisher’s exact test to examine associations between qualitative variables. Sensitivity analyses were conducted to evaluate the robustness of the findings. Anonymized data were handled according to applicable laws and ethical guidelines, ensuring patient privacy and confidentiality. The distribution of quantitative variables was evaluated using the Shapiro-Wilk test. All variables have non-normal distribution. A *p*-value of less than 0.05 was considered statistically significant.

Variables: The key variables of interest included diagnoses, patient characteristics (age, gender, and location), examination data, recommendations made during visits, and visit type (in-person vs. remote). Recommendations were coded into categories for analysis, covering aspects like follow-up, additional tests, specialist consultations, lifestyle interventions, and rehabilitation.

## 3. Results

The results aim to elucidate notable distinctions in chronic disease management between rural and urban patients, alongside the ramifications of the COVID-19 pandemic on these distinctions. Utilizing statistical analyses and a comparative examination of pre-pandemic and pandemic-era data, we offer valuable insights into patient demographics, visit attributes, and diagnosis patterns, as delineated in Table 1, Table 2, Table 3 and Table 4.

The Wilcoxon test revealed a significant difference in age between rural and urban patients (*p* < 0.001), with rural patients having a higher median age (64 years) compared to urban patients (62 years).

However, the Fisher’s test found no significant difference in gender distribution between rural and urban patients (*p* = 0.626). Despite similar gender distributions, rural patients were generally older than urban patients.

Additionally, the Wilcoxon test indicated a significant difference in the number of patient visits between rural and urban patients (*p* < 0.001), with rural patients having a higher median number of visits (seven) compared to urban patients (two). Moreover, age at rural visits was significantly higher than at urban visits (*p* < 0.001), with respective medians of 68 and 65.0 years.

Furthermore, the Fisher’s test showed a significant association between gender distribution at visits and the center’s location, with a higher percentage of women at rural visits than urban visits.

Lastly, the distribution of major diagnoses (E10, E11, I10, I11) and visit types also differed significantly between rural and urban centers (*p* = 0.002 and *p* < 0.001, respectively), with urban centers exhibiting a higher prevalence of specific diagnoses and outpatient visits. In comparison, rural centers had a higher prevalence of remote visits.

The Wilcoxon test revealed a significant difference in age at visits between the two COVID periods (*p* < 0.001), with a higher median age during the COVID period compared to the pre-COVID period (68 years vs. 66 years).

However, itisimportant to note that this difference may partly result from a simple shift in timing between the periods, although the influence of the pandemic cannot be discounted.

On the other hand, the Fisher’s test found no significant difference in gender distribution at visits between the pre-COVID and COVID periods (*p* = 0.374), with similar percentages of women and men during both periods.

However, there was a significant difference in the distribution of diagnoses at visits between the two periods (*p* < 0.001), with a higher prevalence of specific diagnoses during the pre-COVID period, particularly for E10, E11, and I11 diagnoses.Furthermore, the distribution of visit types during the COVID period showed a significant difference (*p* < 0.001), with a higher percentage of outpatient visits during the pre-COVID period compared to the COVID period (100% vs. 42.4%) and a higher percentage of remote visits during the COVID period (57.6% vs. 0%).

The Wilcoxon test showed no significant age differences between patients at rural and urban visits (*p* = 0.27), indicating similar ages during the COVID and pre-COVID periods (Table 4). However, there is a notable difference in the gender distribution between the two periods. Pre-COVID visits had a higher percentage of women thanCOVID visits (32.2% vs. 9.5%). In contrast, the opposite was observed for COVID visits, with a higher percentage of women than pre-COVID visits (90.5% vs. 67.8%).Furthermore, the Fisher’s test revealed a significant difference in the distribution of visit types between the COVID and pre-COVID periods (*p* < 0.001).

Pre-COVID visits had a higher percentage of outpatient visits than the COVID period (100% vs. 39.2%).Similarly, in another analysis, the Wilcoxon test showed no significant age differences between patients at rural and urban visits (*p* = 0.43) during both COVID and pre-COVID periods.

Additionally, the Fisher’s test found no significant difference in gender distribution during the COVID period (*p* = 0.239), indicating similar gender distributions across both periods. However, there was a substantial difference in the distribution of visit types between the COVID and pre-COVID periods (*p* < 0.001), with a higher percentage of outpatient visits during the pre-COVID period compared to the COVID period (100.0% vs. 41.2%)

The Wilcoxon test revealed significant age differences at COVID visits (*p* < 0.001), with the median age higher at COVID visits compared to pre-COVID visits (65 vs. 61 years). Conversely, the Fisher’s test showed no significant difference in gender distribution during the COVID periods (*p* = 0.906), indicating similar gender distributions across both periods.

Furthermore, the Fisher’s test demonstrated a significant difference in the distribution of visit types between the COVID and pre-COVID periods (*p* < 0.001). Pre-COVID visits had a higher percentage of outpatient visits than the COVID period (100% vs. 47.5%). In comparison, remote visits were more prevalent during the COVID period compared to the pre-COVID period (52.5% vs. 0%).

The Wilcoxon test rejected the hypothesis of no age differences at COVID visits in the study population (*p* < 0.001). The median age at COVID visits is higher than pre-COVID visits, respectively 81 and 74 years.The Fisher’s test rejected the hypotheses of independence of the gender distribution on visits from COVID periods in the study population (*p* < 0.001).

We observe a higher percentage of women at visits during the COVID period than during the pre-COVID period, respectively: 68.1% and 62.0%.The Fisher’s test rejected the hypothesis of independence of the distribution of the type visits from the COVID period in the study population (*p* < 0.001). We observe a higher percentage of outpatient visits during the pre-COVID period than during the COVID period: 100% vs. 29%. We observe a higher percentage of remote visits during the COVID period than during the pre-COVID period, respectively:71% vs 0%.

**Figure 1:**
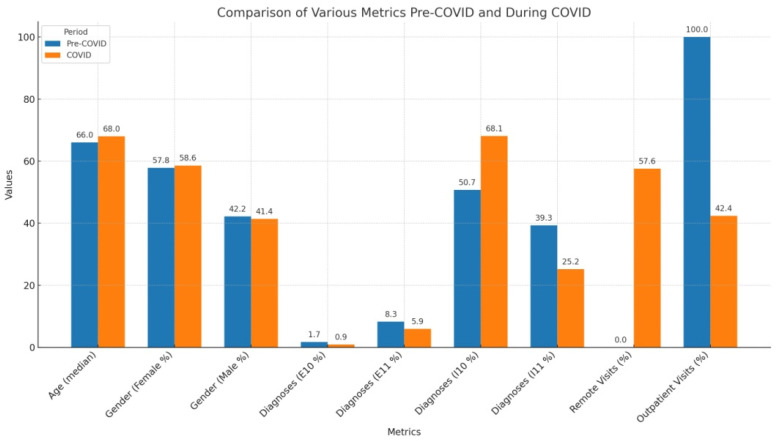
Central figure of the results.

The below Figure 1 summarizes the results.

## 4. Discussion

Data from 2018 to the first quarter 2021 were analyzed to identify unique challenges and opportunities in managing these conditions across different settings. The study provides insights into enhancing chronic disease management by examining patient visits and recommendations, particularly in light of the COVID-19 pandemic’s impact on healthcare delivery and patient outcomes.

Current health policies emphasize the management of chronic conditions, which cannot be cured but can be controlled through medication, therapy, and lifestyle changes to prevent complications [21,43,44,45]. However, translating evidence-based guidelines into everyday medical practice faces challenges, with a limited understanding of barriers and facilitators hindering implementation. Engaging patients and caregivers is crucial for effective interventions, with a need for tailored, context-specific approaches, including personalized medicine strategies to optimize outcomes.

Effective prevention and treatment of chronic diseases like hypertension and diabetes require comprehensive, multi-level interventions, although overly rapid or complex innovations can impede progress [21,43,44,45]. Regular monitoring and evaluation, with defined outcomes and indicators, are essential for successful program implementation across diverse health contexts. It is vital to bridge the gap between evidence-based guidelines and clinical practice through flexible, culturally sensitive, patient-centered approaches to improve health outcomes.

The prevention and management of chronic conditions are critical in global healthcare. Still, evidence-based guidelines often face challenges in implementation, with patient adherence and guideline overload in general practice being notable issues [46,47]. Engaging patients and their local environments in decision-making is essential for effective healthcare management, requiring tailored approaches across diverse contexts.

The COVID-19 pandemic significantly influenced primary healthcare delivery, with a notable shift toward telemedicine observed during the pandemic [10]. The pandemic highlighted the importance of adaptation and innovation in healthcare delivery, particularly in accessing post-diagnosis care and support [4,5]. Gender disparities in healthcare-seeking behavior were evident during the COVID period, with further research needed to explore underlying reasons [4,5].

Rural patients tend to have a higher average age and more visits than urban patients, suggesting potential differences in healthcare needs and utilization patterns influenced by cultural and socioeconomic factors [4,5]. Urban medical centers showed a higher prevalence of specific diagnoses like diabetes and hypertension, possibly due to factors like population density and lifestyle differences [4,5]. Understanding these disparities is crucial for developing effective healthcare policies and interventions to address the needs of diverse patient populations.

To effectively prevent and manage chronic diseases, healthcare providers must have the knowledge and skills to deliver tailored, evidence-based care [46,47]. However, translating guidelines into practice can be challenging, particularly in resource-limited or non-standard settings. Training programs are vital in scaling best practices and promoting patient-centered care [46,47]. The theoretical model of adaptive implementation provides a framework for designing and implementing health interventions that can be adapted over time to fit different contexts and populations [48,49,50]. Dröes’s model emphasizes flexibility and adaptation in implementing evidence-based interventions, recognizing implementation as a continuous process [49,50]. Barriers to implementation may arise at multiple levels of healthcare delivery, including patient, provider, organizational, and policy levels [49,50]. Guidelines should be evidence-based, broad-based, flexible, and culturally acceptable, with stakeholder involvement crucial for their effectiveness [21].

An evidence-based approach to prevention is crucial for minimizing the burden of chronic diseases [51,52]. To achieve this, there is a need for evidence derived from complex intervention evaluation methodologies in diverse health and social care contexts [52]. Integrated care, facilitated by proactive healthcare teams and functional information exchange networks, is criticalto improving the quality of care [51]. Individualized support for patients in making lifestyle changes for prevention can lead to improved outcomes, but cultural factors may pose challenges to effective implementation [51]. Recommendations for enhancing the evaluation of integrated care include implementing research methods, refining data collection methods for vulnerable populations, and prioritizing functional information exchange networks [52]. Addressing social determinants of health, such as poverty and access to care, is essential for improving diabetes care outcomes in socially disadvantaged populations. Collaboration among professionals and organizations, increased awareness of healthy lifestyle recommendations, and personalized prevention programs are necessary approaches to strengthen chronic disease prevention [52]. Coordinated approaches to health policies and programs, along with adequate funding and support for social policies, can contribute to better prevention outcomes [52]. Understanding the mechanisms through which public health interventions are scaled up is crucial for their effective implementation on a broader scale.

### 4.1. Recommendations for Clinical Practice

Interpreting our findings in the context of clinical practice underscores the imperative for general practitioners to address the nuanced differences in chronic disease management between rural and urban populations. Our study illuminates the impact of the COVID-19 pandemic on these disparities, necessitating adaptive strategies to ensure continuity of care.

In light of the complexity of multimorbidity, it becomes paramount for healthcare providers to prioritize patient-centric approaches, particularly within general practice. Guideline developers should account for these complexities, tailoring interventions to meetpatient needs and preferences.

Future clinical trials should explore the efficacy of composite lifestyle metrics, such as optimal lifestyle scores, in mitigating complications associated with chronic diseases like metabolic syndrome. Understanding the long-term effectiveness of lifestyle interventions can inform evidence-based strategies for sustained behavior change.

Further research on innovative approaches and health technology assessments is warranted to assess their impact on patient care, quality, and cost-effectiveness. This includes rigorous evaluations of implementation strategies to identify best practices for scaling up public health interventions.

Emphasizing patient involvement in designing and evaluating public health programs is crucial for ensuring interventions are culturally sensitive and aligned with patient preferences. Collaboration with patient advocacy groups and community organizations can facilitate tailored interventions that resonate with diverse populations.

Developing training programs that empower healthcare providers to contextualize guidelines and implement patient-centered care strategies is essential for achieving population-wide improvements in chronic disease prevention and management. Education on behavior change strategies and effective intervention implementation can enhance the delivery of personalized care.

By embracing these perspectives, healthcare systems can proactively address the evolving challenges of chronic disease management, ultimately leading to improved health outcomes for individuals and populations.

### 4.2. Study Limitations

Retrospective data analysis has limitations due to reliance on the accuracy and completeness of original records. Only complete, correct, or standardized data can impact quality and limit conclusions. Such studies are observational, establishing associations rather than causality, and may lack relevance to the current context or population if conducted at a different time or place. Additionally, available data may not cover all relevant variables or allow the examination of specific hypotheses.

## 5. Conclusions

The findings revealed significant differences between rural and urban patients regarding age, number of patient visits, gender distribution, and types of diagnoses and visit modalities. Rural patients tended to be older, had a higher average number of visits, and exhibited different patterns of diagnoses and visit types compared to urban patients. These disparities highlight the need for tailored approaches to chronic disease management based on geographic location and patient demographics.

Furthermore, the study investigated the impact of the COVID-19 pandemic on chronic disease treatment. While age at visits increased during the pandemic period, possibly influenced by shifts in healthcare-seeking behavior, there were no significant changes in gender distribution. However, there was a noticeable shift in the distribution of diagnoses and visit modalities, with increased remote visits and changes in the prevalence of specific diagnoses during the pandemic.

The study underscores the importance of understanding the unique challenges and opportunities in managing chronic diseases across different settings and during public health crises like the COVID-19 pandemic. By identifying these factors, healthcare providers and policymakers can develop targeted interventions to improve chronic disease prevention and management, ultimately leading to better health outcomes for individuals and communities. Further research is needed to explore the long-term effects of the pandemic on chronic disease treatment and to assess the effectiveness of interventions aimed at mitigating its impact.

## Figures and Tables

**Table 1:** Target population description.

**Age Distribution of Patients in the Rural and Urban Centers**									
centre	n		age						Wilcoxon test *p*
			median				min	max	
rural	1472		64				7	96	*p* < 0.001
urban	451		62				21	92	
total	1923		63				7	96	
**Gender distribution of patients in the rural and urban centers**									
centre	gender								Fisher test *p*
	female				male				
	n		%		n		%		
rural	816		55.4		656		44.6		0.626
urban	256		56.8		195		43.2		
total	1072		55.7		851		42.3		n = 1923
**Distribution of the number of patient visits in the rural and urban centers**									
centre	n		number of patient visits						Wilcoxon test *p*
			median		min		max		
rural	1472		7		1		36		*p* < 0.001
urban	451		2		1		25		
total	1923		6		1		36		
**Age distribution on visits to rural and urban centers**									
centre	n		age						Wilcoxon test *p*
			median						
rural	12,591		68						*p* < 0.001
urban	1242		65						
total	13,833		67						
**Gender distribution on visits to rural and urban centers**									
centre	gender								Fisher test *p*
	female				male				
	n		%		n		%		
rural	7390		58.7		5201		41.3		0.002
urban	672		54.1		570		45.9		
total	8062		58.3		5771		41.7		13,833
**Distribution of the presence of major diagnoses (E10, E11, I10, I11) on visits to rural and urban centers**									
centre	E10		E11		I10		I11		Fisher test *p*
	n	%	n	%	n	%	n	%	
rural	86	0.7	669	5.3	7660	60.8	4176	33.2	*p* = 0.002
urban	78	6.3	270	21.7	850	68.4	44	3.5	
total	164	1.2	939	6.8	8510	61.5	4220	30.5	13,833
**Distribution of the type of visits in the rural and urban centers**									
centre	visit’s type								Fisher test *p*
	remote				outpatient				
	n		%		n		%		
rural	4742		37.7		7849		62.3		*p* < 0.001
urban	221		17.8		1021		82.2		
total	4963		35.9		8870		64.1		

**Table 2:** Impact of COVID-19 period on chronic disease.

**Age Distribution at Visits during the COVID Period**									
period	n		age						Wilcoxon test *p*
			median						
pre-COVID	5220		66						*p* < 0.001
COVID	8613		68						
total	13,833		67						
**Gender distribution on visits during the COVID period**									
period	gender								Fisher test *p*
	female				male				
	n		%		n		%		
pre-COVID	3017		57.8		2203		42.2		0.374
COVID	5045		58.6		3568		41.4		
total	8062		58.3		5771		41.7		13,833
**Distribution of the presence of the main diagnoses (E10, E11, I10, I11) at visits during the COVID periods**									
period	E10		E11		I10		I11		Fisher test
	n	%	n	%	n	%	n	%	*p*
pre-COVID	90	1.7	432	8.3	2648	50.7	2050	39.3	*p* < 0.001
COVID	74	0.9	507	5.9	5862	68.1	2170	25.2	
total	164	1.2	939	6.8	8510	61.5	4220	30.5	13,833
**Distribution of the type of visit in the COVID period**									
period	visit’s type								Fisher test *p*
	remote				outpatient				
	n		%		n		%		
pre-COVID	0		0.0		5220		100.0		*p* < 0.001
COVID	4963		57.6		3650		42.4		
total	4963		35.9		8870		64.1		

**Table 3:** Visits with E10 and E11 diagnosis:analysis of the pre-COVID vs. COVID period.

**cE10—Age Distribution at Visits during the COVID Periods**					
E10 period	n	age			Wilcoxon test *p*
		median			
pre-COVID	90	55.0			0.27
COVID	74	52.5			
total	164	55			
**cE10—Gender distribution at visits during the COVID periods**					
E10 period	gender				Fisher test *p*
	female		male		
	n	%	n	%	
pre-COVID	29	32.2	61	67.8	*p* < 0.001
COVID	7	9.5	67	90.5	
total	36	22.0	128	78.0	
**cE10—Distribution of the type of visits during the COVID periods**					
E10 period	visit’s type				Fisher test *p*
	remote		outpatient		
	n	%	n	%	
pre-COVID	0	0.0	90	100.0	0
COVID	45	60.8	29	39.2	
total	45	27.4	119	72.6	
**cE11—Age distribution at visits during the COVID periods**					
E11 period		age			Wilcoxon test *p*
	n	median			
pre-COVID	432	66			0.43
COVID	507	65			
total	939	65			
**cE11—Gender distribution at visits during the COVID periods**					
E11 period	gender				Fisher test *p*
	female		male		
	n	%	n	%	
pre-COVID	218	50.5	214	49.5	0.239
COVID	236	46.5	271	53.5	
total	454	48.3	485	51.7	
**cE11—Gender distribution at visits in the COVID periods**					
E11 period	visit’s type				Fisher test *p*
	remote		outpatient		
	n	%	n	%	
pre-COVID	0	0.0	432	100.0	*p* < 0.001
COVID	298	58.8	209	41.2	
total	298	31.7	641	68.3	

**Table 4:** Visits with I10 and I11 diagnosis: analysis of pre-COVID vs. COVID periods.

**cI10—Age Distribution at Visits during the COVID Periods**					
I10 period	n	age			Wilcoxon test
		median			
pre-COVID	2648	61			*p* < 0.001
COVID	5862	65			
total	8510	64			
**cI10—Gender distribution at visits during the COVID periods**					
I10 period	gender				Fisher test *p*
	Female		male		
	n	%	n	%	
pre-COVID	1498	56.6	1150	43.4	0.906
COVID	3325	56.7	2537	43.3	
total	4823	56.7	3687	43.3	
**cI10—Distribution of the type of visits during the COVID periods**					
I10 period	visit’s type				Fisher test *p*
	Remote		outpatient		
	n	%	n	%	
pre-COVID	0	0.0	2648	100.0	*p* < 0.001
COVID	3080	52.5	2782	47.5	
total	3080	36.2	5430	63.8	
**cI11—Age distribution at visits during the COVID periods**					
I11 period	n	age			Wilcoxon test *p*
		median			
pre-COVID	2050	74			*p* < 0.001
COVID	2170	81			
total	4220	78			
**cI11—Gender distribution at visits during the COVID periods**					
I11 period	gender				Fisher test *p*
	Female		male		
	n	%	n	%	
pre-COVID	1272	62.0	778	38.0	*p* < 0.001
COVID	1477	68.1	693	31.9	
total	2749	65.1	1471	34.9	
**cI11—Distribution of the type of visits during the COVID periods**					
I11 period	visit’s type				Fisher test *p*
	Remote		outpatient		
	n	%	n	%	
pre-COVID	0	0	2050	100	*p* < 0.001
COVID	1540	71	630	29	
total	1540	36.5	2680	63.5	

## Data Availability

The original contributions presented in the study are included in the article. Further inquiries can be directed to the corresponding author.
